# Analysis of risk factors for early neurological deterioration after intravenous thrombolysis in patients with acute ischemic stroke

**DOI:** 10.3389/fneur.2025.1555708

**Published:** 2025-08-05

**Authors:** Jungang Fang, Hui Sun, Xinyu Lu

**Affiliations:** ^1^People's Hospital Affiliated to Jiangsu University, School of Medicine Jiangsu University, Zhenjiang, China; ^2^Hospital Affiliated to Jiangsu University, Zhenjiang, China

**Keywords:** acute ischemic stroke, intravenous thrombolysis, early neurological deterioration, NIHSS score, TOAST

## Abstract

**Objective:**

The aim of this study is to examine the potential risk factors contributing to early neurological deterioration (END) following intravenous thrombolysis in patients diagnosed with acute ischemic stroke (AIS).

**Methods:**

Clinical data was gathered from AIS patients who underwent intravenous thrombolysis at the Affiliated People’s Hospital of Jiangsu University between January 2020 and December 2023. Patients were subsequently categorized into two groups based on the occurrence of END within 24 h post-thrombolysis: the END group and the non-END group. A comparative analysis of the clinical data from both groups was conducted. The application of Multivariate Logistic regression facilitated the identification of independent risk factors and the construction of a nomogram prediction model.

**Results:**

The study encompassed a total of 249 AIS patients, with 32 patients (12.9%) developing END. The multivariate analysis revealed that NIHSS Score immediately after thrombolysis (*p* < 0.001), the Trial of Org 10172 in Acute Stroke Treatment (TOAST) typing of large artery atherosclerosis (*p* = 0.025), and a prior history of diabetes (*p* = 0.023) were independent risk factors for the development of END post-thrombolysis. A nomogram was constructed to generate the ROC curve, and the AUC value was 0.809 (95% CI, 0.732–0.885).

**Conclusion:**

This nomogram, which demonstrates good discrimination and high predictive value, identifies NIHSS score immediately after thrombolysis, TOAST classification of large artery atherosclerosis, and a history of diabetes as independent risk factors for early neurological deterioration (END) in acute ischemic stroke (AIS) patients receiving intravenous thrombolysis.

## Introduction

Acute ischemic stroke (AIS) is a disease caused by cerebral vascular occlusion, which leads to ischemia and hypoxia of brain tissue and necrosis. It has a high morbidity, disability and mortality rate, accounting for 70–80% of all strokes, seriously endangering human health and causing a huge socioeconomic burden (1, 2). Reperfusion, including intravenous thrombolysis (IVT) and intravascular therapy within the designated time window, is the cornerstone of treatment for acute ischemic stroke (AIS). It aims to reopen occluded blood vessels, restore blood flow, and salvage brain tissue in the ischemic penumbra. Intravenous thrombolysis, with its advantages of simplicity and cost-effectiveness, remains a primary reperfusion strategy for AIS (3). While intravenous thrombolysis effectively alleviates symptoms in most patients, a subset experiences early neurological deterioration (END), characterized by worsening neurological deficits.

The occurrence of early neurological deterioration (END) may be mechanistically attributed to both hemodynamic instability and systemic metabolic dysregulation, wherein the former encompasses critical vascular pathologies including persistent arterial occlusion and insufficient collateral circulation that collectively contribute to diminished cerebral perfusion pressure, while the latter involves complex pathophysiological processes characterized by ischemia-induced neurotoxicity and hypoxia-triggered inflammatory cascades that synergistically exacerbate secondary brain injury. Current evidence identifies several clinically significant predictors of END development, particularly advanced age, elevated admission blood glucose levels, large artery atherosclerosis, and internal carotid artery occlusion; however, the predictive validity and clinical utility of these biomarkers remain incompletely validated and warrant further rigorous investigation through large-scale prospective studies to establish their prognostic significance and potential therapeutic implications in acute stroke management (4–8). This study aims to investigate the risk factors associated with END in AIS patients following thrombolysis and to develop a nomogram that can serve as a valuable tool for guiding clinical stroke management.

## Methods

### Study design and participants

The data of AIS patients who received intravenous thrombolysis at the Affiliated People’s Hospital of Jiangsu University from January 2020 to December 2023 were retrospectively analyzed. Inclusion criteria: (1) age > 18 years; (2) admission within 4.5 h after onset; (3) patients signed the informed consent for intravenous thrombolysis. (4) treated with intravenous thrombolysis. Exclusion criteria: (1) imaging showed cerebral hemorrhage after IVT; (2) patients with large vessel occlusion received intravascular treatment. This study was approved by the Ethics Committee of the Affiliated People’s Hospital of Jiangsu University. Patient informed consent for inclusion in this study was waived.

### Treatment methods

AIS patients meeting the inclusion criteria received intravenous thrombolysis with recombinant tissue plasminogen activator (rtPA). The dosage was 0.9 mg/kg, with a maximum dose of 90 mg. The initial 10% of the dose was administered as a bolus injection, followed by a continuous intravenous infusion over 1 h. Vital signs and neurological function were monitored closely throughout treatment and for 24 h after thrombolysis.

### Criteria for END

The NIHSS score was used to evaluate the neurological deficit of AIS patients. NIHSS assessments were performed at three timepoints: upon admission (pre-thrombolysis), immediately after completion of thrombolysis, and at 24 h post-thrombolysis. END was defined as an increase of ≥4 points in the NIHSS score at 24 h compared with the baseline (pre-thrombolysis), or death within 24 h of treatment. Cases where deterioration was causally linked to imaging-confirmed intracranial hemorrhage (per exclusion criterion #1) were excluded from END classification, as these represent distinct pathophysiological entities.

### Data acquisition

Upon admission, patient demographics including age, sex, BMI, medical history (diabetes, hypertension, coronary heart disease, atrial fibrillation), time from admission to thrombolysis, acute ischemic stroke subtypes based on the Trial of Org 10172 in Acute Stroke Treatment (TOAST) classification were recorded. NIHSS scores were recorded at three timepoints: pre-thrombolysis, immediately after thrombolysis, and at 24 h post-thrombolysis. Additionally, laboratory tests were performed at admission to assess C-reactive protein, white blood cell count, neutrophil-to-lymphocyte ratio, Neutrophils, lymphocytes, platelet count, uric acid, creatinine, and urea nitrogen.

### Statistical methods

Data were analyzed using SPSS 25.0. For continuous variables with normal distribution, mean ± standard deviation was used and compared using the *t*-test. For skewed distribution, median and interquartile range were reported and compared using the Mann–Whitney U test. Categorical variables were presented as counts and percentages and compared using the chi-square test. Baseline characteristics between the END and non-END groups were compared using univariate analysis. Binary logistic regression analysis was employed. Variables demonstrating statistically significant associations in univariate analyses were subsequently incorporated into a multivariable logistic regression model. This model identified independent risk factors for Early Neurological Deterioration (END). Based on the results of the multivariable analysis, a clinical prediction model was constructed and visualized using a nomogram. The model’s performance was rigorously evaluated across key dimensions: discrimination was assessed using the Receiver Operating Characteristic (ROC) curve and its associated Area Under the Curve (AUC), calibration was examined via calibration plots, and clinical utility was evaluated through Decision Curve Analysis (DCA). Statistical significance was defined as a two-sided *p* value < 0.05. The nomogram prediction model was developed utilizing R software (version 3.3.2; R Foundation for Statistical Computing, Vienna, Austria).

## Results

### Baseline characteristics

This study included 249 AIS patients (88 women and 161 men) aged 30 to 92 years. Of these, 32 (12.9%) experienced END. Based on the performance of END, 249participants were divided into 2groups: END group (*n* = 32) and non- END group (*n* = 217). Compared to the non-END group, the NIHSS score in the END group was significantly higher following thrombolysis (16 vs. 4, *p* < 0.001). Additionally, significant differences were observed between the groups in terms of pre-thrombolysis NIHSS score, TOAST classification, history of diabetes, and serum glucose levels (*p* < 0.05). However, no significant differences were found between the two groups regarding age, admission blood pressure, BMI, history of hypertension, CRP, or white blood cell count (*p* > 0.05). The characteristics of the participants are summarized in Table 1.

**Table 1:** Baseline characteristics of participants.

Variables	Non-END (*n* = 217)	END (*n* = 32)	Statistic	*p*
Demographic characteristics				
Age, M (Q₁, Q₃)	68.00 (58.00, 76.00)	72.50 (59.50, 80.25)	*Z* = −1.55	0.122
Male, *n* (%)	145 (66.82)	16 (50.0)	*χ*^2^ = 6.26	0.063
BMI, M (Q₁, Q₃)	23.88 (22.03, 25.95)	23.63 (21.98, 25.11)	*Z* = −0.44	0.657
Hypertension, *n* (%)	161 (74.19)	23 (71.88)	*χ*^2^ = 0.08	0.780
Diabetes, *n* (%)	50 (23.04)	14 (43.75)	*χ*^2^ = 6.26	0.012
Coronary disease, *n* (%)	30 (13.82)	2 (6.25)	*χ*^2^ = 0.83	0.362
Atrial fibrillation, *n* (%)	24 (11.06)	4 (12.50)	*χ*^2^ = 0.00	1.000
Stroke, *n* (%)	61 (28.11)	12 (37.50)	*χ*^2^ = 1.19	0.276
Clinical assessment				
SBP, M (Q₁, Q₃)	157.00 (140.00, 174.00)	159.50 (141.25, 185.25)	*Z* = −0.04	0.971
DBP, Mean ± SD	91.72 ± 15.75	89.00 ± 19.31	*t* = 0.88	0.378
NIHSS (pre-thrombolysis)			–	0.023
Mild (NIHSS<5)	85 (39.17)	5 (15.62)		
Moderate (5 ~ 20)	107 (49.31)	20 (62.50)		
Severe (NIHSS>20)	25 (11.52)	7 (21.88)		
NIHSS (immediately after thrombolysis), M (Q₁, Q₃)	4.00 (2.00, 10.00)	16.00 (6.00, 24.25)	*Z* = −4.57	<0.001
DNT, M (Q₁, Q₃)	45.00 (31.00, 56.00)	41.50 (33.50, 60.25)	*Z* = −0.22	0.827
TOAST			–	<0.001
SAA	144 (66.36)	11 (34.38)		
LAA	49 (22.58)	17 (53.12)		
CE	21 (9.68)	2 (6.25)		
SOE or SUE	3 (1.38)	2 (6.25)		
Laboratory data				
CRP, M (Q₁, Q₃)	1.55 (0.61, 3.00)	1.36 (0.61, 2.84)	*Z* = −0.26	0.797
WBC, M (Q₁, Q₃)	6.6 (5.6, 8.4)	7.4 (5.9, 9.4)	*Z* = −1.17	0.242
Neutrophil, M (Q₁, Q₃)	4.10 (3.40, 5.40)	4.10 (3.30, 5.50)	*Z* = −0.26	0.793
Lymphocyte, M (Q₁, Q₃)	1.60 (1.20, 2.10)	1.70 (1.10, 2.75)	*Z* = −1.00	0.320
NLR, M (Q₁, Q₃)	2.56 (1.65, 4.20)	2.41 (1.46, 4.19)	*Z* = −0.33	0.740
PLT, M (Q₁, Q₃)	181.00 (144.00, 217.00)	166.50 (150.25, 205.25)	*Z* = −0.78	0.436
Serum glucose, M (Q₁, Q₃)	7.61 (6.34, 10.21)	9.93 (7.29, 11.11)	*Z* = −2.23	0.026
Creatinine, M (Q₁, Q₃)	67.74 (56.74, 84.00)	70.06 (59.61, 82.16)	*Z* = −0.10	0.924
Urea nitrogen, M (Q₁, Q₃)	5.94 (5.05, 7.12)	5.79 (4.99, 7.77)	*Z* = −0.10	0.923
Uric acid, M (Q₁, Q₃)	325.45 (261.74.00, 392.31)	341.06 (255.97, 413.00)	*Z* = −0.49	0.623

BMI, body mass index; SBP, systolic blood pressure; DBP, diastolic blood pressure; NIHSS, National Institute of Health stroke scale; DNT, Door-to-Needle Time; TOAST, Trial of Org 10172 in Acute Stroke Treatment; SAA, Small-artery occlusion lacunar; LAA, Large-artery atherosclerosis; CE, Cardioembolism; SOE, Stroke of other determined cause; SUE, Stroke of undetermined cause; CRP, C-reactive protein; WBC, white blood cell; NLR, Neutrophil-to-Lymphocyte Ratio.

### Univariate and multivariate analysis

Multivariate analysis was performed to identify predictors for END after intravenous thrombolysis. The results are presented in Table 2. The significant factors in the univariate analysis were included in the multivariate logistic regression analysis, which showed that a higher NIHSS Score immediately after thrombolysis (*p* < 0.001), diabetes (*p* = 0.023), and TOAST subtype of large arterial atherosclerosis (LAA) (*p* = 0.025) was independently positively associated with END occurrence in AIS patients after thrombolysis.

**Table 2:** Risk parameters of END in AIS patients after thrombolytic therapy based on univariate and multivariate analysis.

Variables	Univariate					Multifactorial				
	*β*	S. E	*Z*	*p*	OR (95%CI)	*β*	S. E	*Z*	*p*	OR (95%CI)
Age	0.03	0.02	1.58	0.113	1.03 (0.99 ~ 1.06)					
BMI	−0.02	0.05	−0.34	0.733	0.98 (0.89 ~ 1.09)					
SBP	−0.00	0.01	−0.35	0.726	1.00 (0.99 ~ 1.01)					
DBP	−0.01	0.01	−0.88	0.376	0.99 (0.97 ~ 1.01)					
DNT	0.00	0.01	0.31	0.753	1.00 (0.99 ~ 1.02)					
NIHSS (pre-thrombolysis)										
Mild (NIHSS<5)					1.00 (Reference)					1.00 (Reference)
Moderate (5 ~ 20)	1.16	0.52	2.22	0.026	3.18 (1.15 ~ 8.82)	−0.02	0.65	−0.04	0.970	0.98 (0.28 ~ 3.46)
Severe (NIHSS>20)	1.56	0.63	2.48	0.013	4.76 (1.39 ~ 16.30)	−1.84	1.14	−1.61	0.106	0.16 (0.02 ~ 1.48)
NIHSS (immediately after thrombolysis)	0.09	0.02	4.73	<0.001	1.09 (1.05 ~ 1.13)	0.13	0.04	3.78	<0.001	1.14 (1.07 ~ 1.23)
TOAST										
SAA					1.00 (Reference)					
LAA	1.36	0.39	3.48	<0.001	3.89 (1.81 ~ 8.34)	0.98	0.43	2.25	0.025	2.65 (1.13 ~ 6.22)
CE	−0.47	0.77	−0.62	0.535	0.62 (0.14 ~ 2.79)					
SOE or SUE	1.56	0.93	1.67	0.095	4.76 (0.76 ~ 29.63)					
Hypertension										
NO					1.00 (Reference)					
YES	−0.12	0.42	−0.28	0.781	0.89 (0.39 ~ 2.04)					
Diabetes										
NO					1.00 (Reference)					1.00 (Reference)
YES	0.95	0.39	2.44	0.015	2.60 (1.21 ~ 5.59)	1.00	0.44	2.27	0.023	2.71 (1.15 ~ 6.39)
Coronary disease										
NO					1.00 (Reference)					
YES	−0.88	0.76	−1.16	0.246	0.42 (0.09 ~ 1.83)					
Atrial fibrillation										
NO					1.00 (Reference)					
YES	0.14	0.58	0.24	0.810	1.15 (0.37 ~ 3.56)					
Stroke										
NO					1.00 (Reference)					
YES	0.43	0.40	1.08	0.279	1.53 (0.71 ~ 3.33)					
CRP	−0.02	0.04	−0.45	0.654	0.98 (0.90 ~ 1.07)					
WBC	0.10	0.08	1.27	0.205	1.10 (0.95 ~ 1.28)					
Neutrophil	0.04	0.08	0.49	0.623	1.04 (0.88 ~ 1.23)					
Lymphocyte	0.22	0.18	1.28	0.202	1.25 (0.89 ~ 1.76)					
NLR	−0.02	0.07	−0.24	0.810	0.98 (0.85 ~ 1.14)					
PLT	−0.00	0.00	−0.19	0.850	1.00 (0.99 ~ 1.01)					
Uric acid	0.00	0.00	0.47	0.640	1.00 (1.00 ~ 1.00)					
Serum glucose	0.07	0.04	1.81	0.070	1.07 (0.99 ~ 1.16)					
Creatinine	−0.00	0.01	−0.30	0.763	1.00 (0.98 ~ 1.01)					
Urea nitrogen	0.02	0.10	0.15	0.879	1.02 (0.84 ~ 1.23)					

### Construction and validation of the nomogram

**Figure 1:**
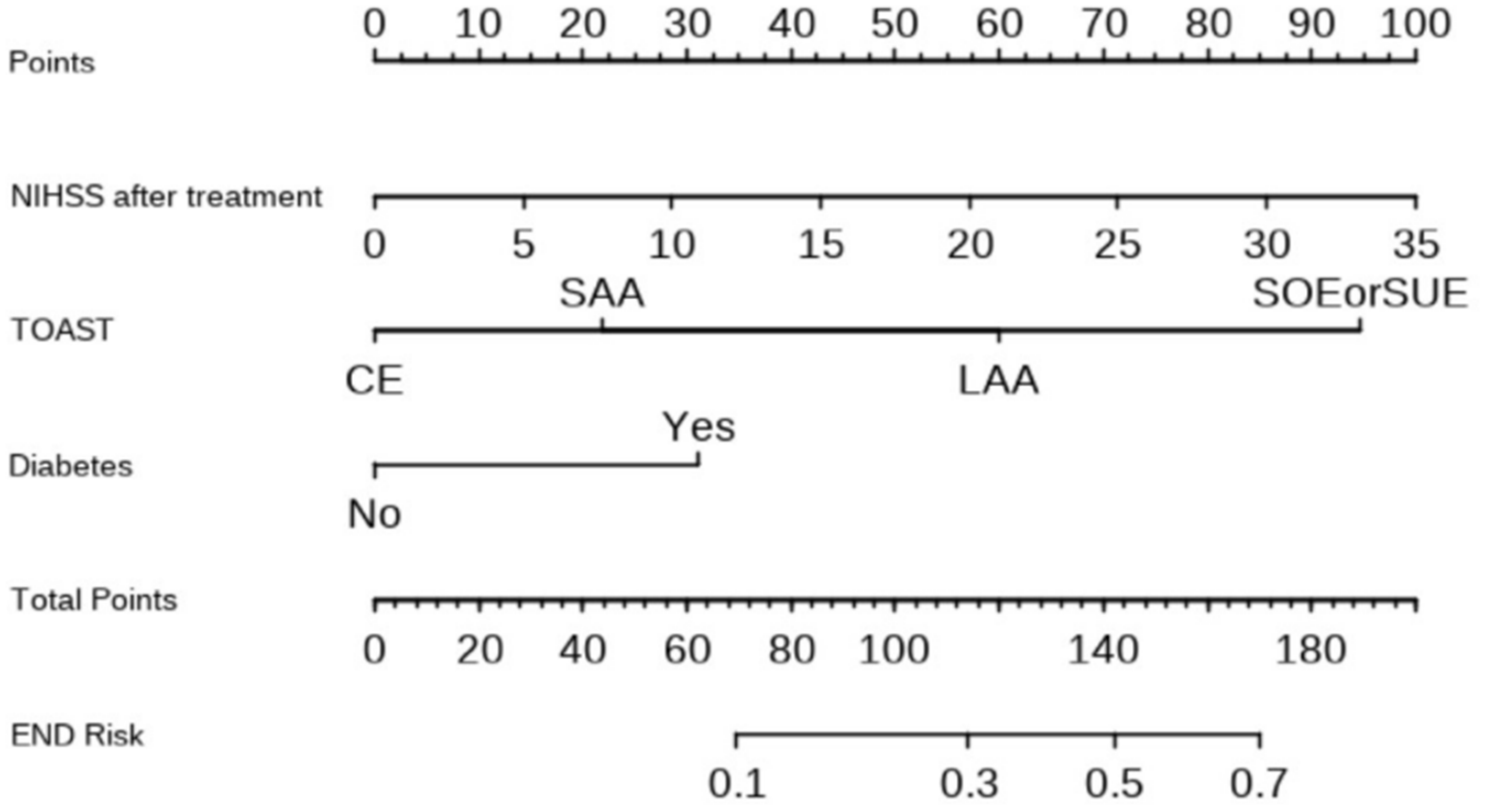
Nomogram for predicting the risk of END in patients after thrombolysis. Each of the three indicator scores is aligned with the “Points” line and then the three scores are added together to yield “Total Points,” which can then be used to predict END risk.

Based on these findings, a nomogram prediction model for END in AIS patients after thrombolysis was constructed (Figure 1). This nomogram assigns a score to each predictive indicator, allowing for a cumulative total score to be calculated. The total score is then converted into a corresponding predictive probability, providing an estimate of the risk of END following thrombolysis.

**Figure 2:**
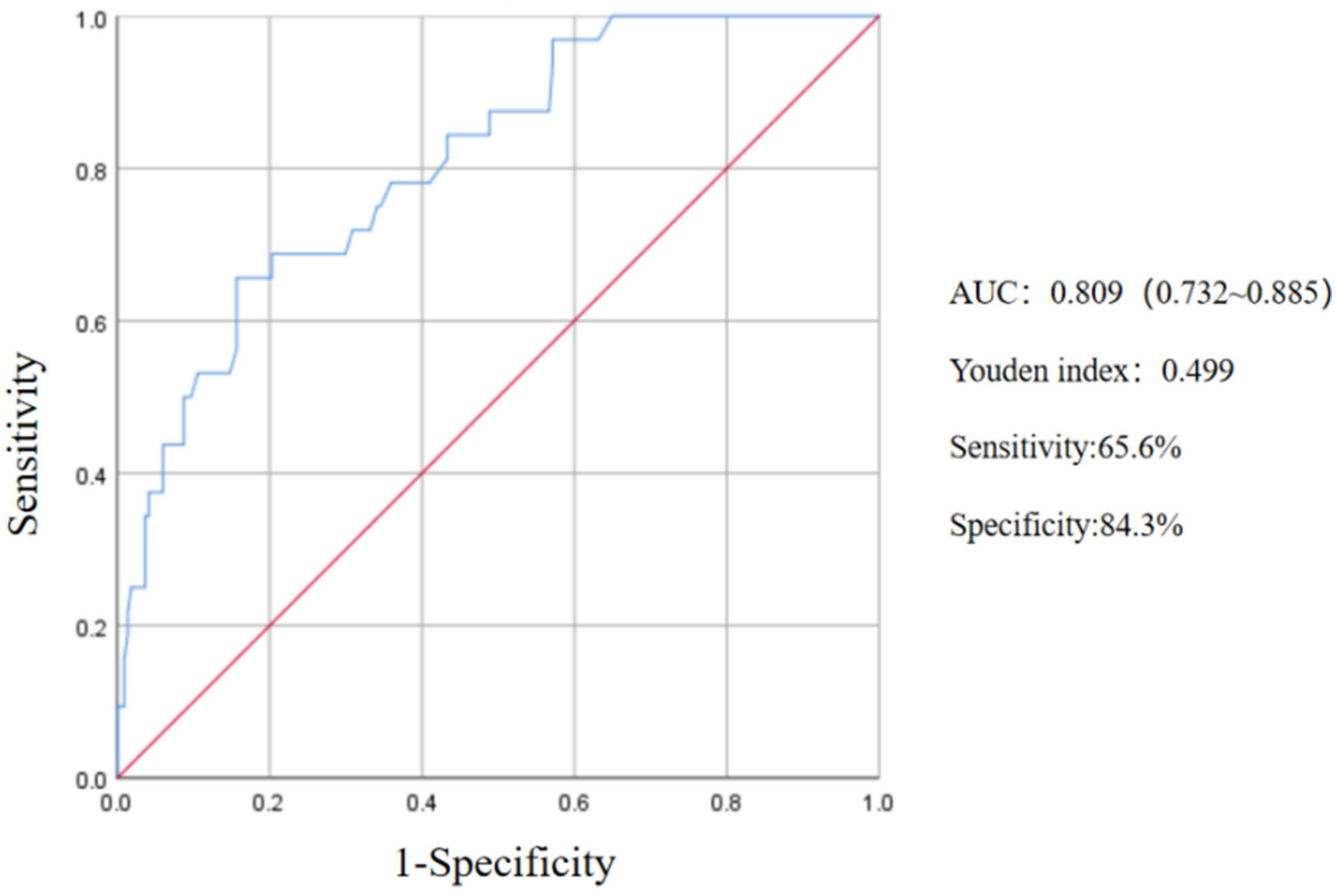
ROC curve of the predictive value of NIHSS score, TOAST and diabetes on END after thrombolysis.

The Receiver Operating Characteristic (ROC) curve was constructed by integrating the NIHSS Score immediately after thrombolysis, the TOAST classification for stroke, and the diabetes disease index following thrombolysis. The analysis revealed an area under the ROC curve of 0.809 (95% CI, 0.732–0.885, *p* < 0.05). The maximum Youden index was determined to be 0.499, indicating a sensitivity of 65.6% and a specificity of 84.3%, thereby demonstrating a notable predictive value (Figure 2).

**Figure 3:**
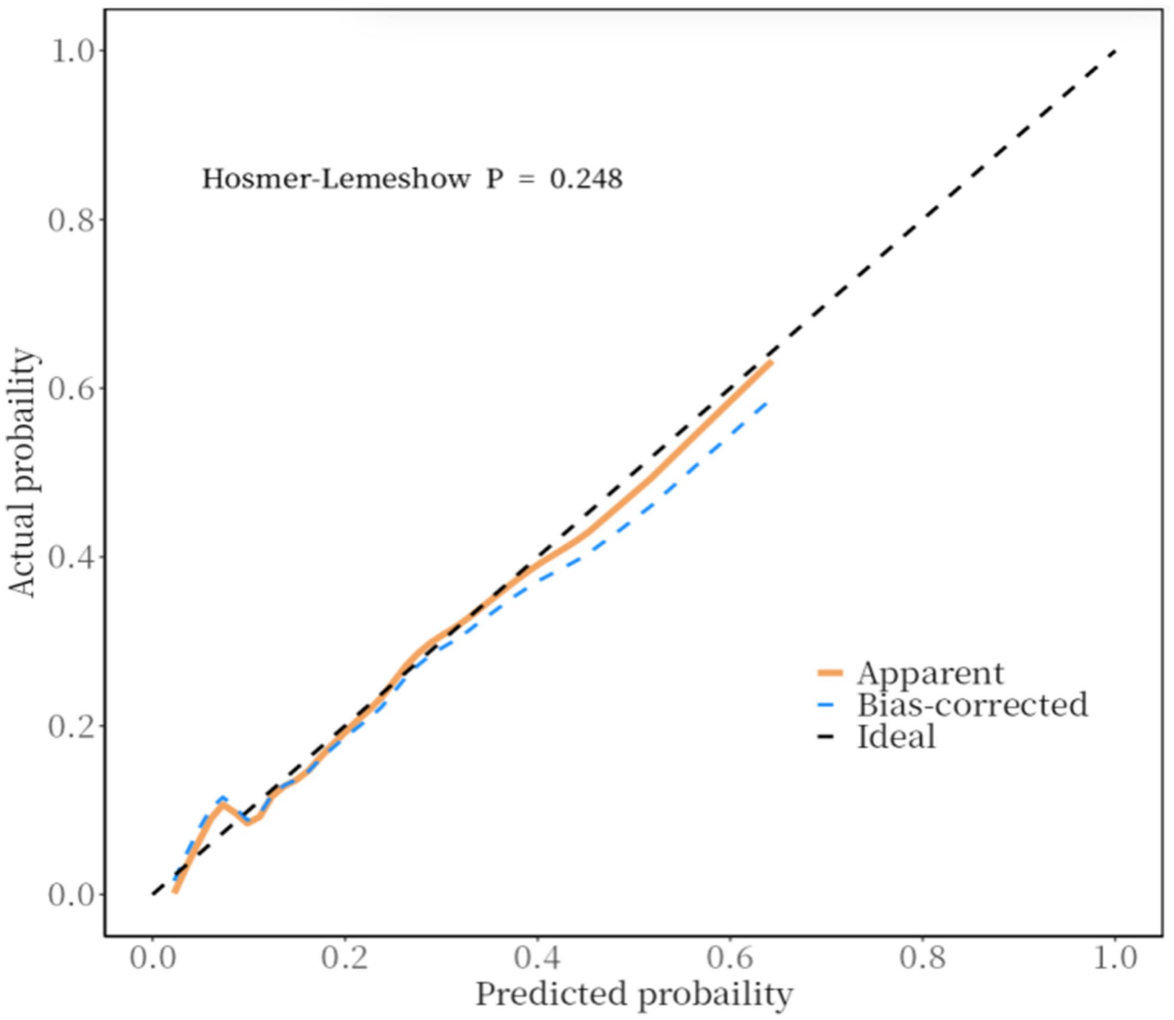
END clinical prediction model calibration curve.

Furthermore, a calibration curve was constructed to assess the calibration accuracy of the predictive model. The findings indicated that both the logistic and nonparametric calibration curves exhibited minimal deviation from the ideal curve. The Hosmer-Lemeshow goodness-of-fit test yielded a *χ*^2^ value of 10.243 with a *p*-value of 0.248, suggesting that the predictive accuracy of the model is satisfactory (Figure 3).

**Figure 4:**
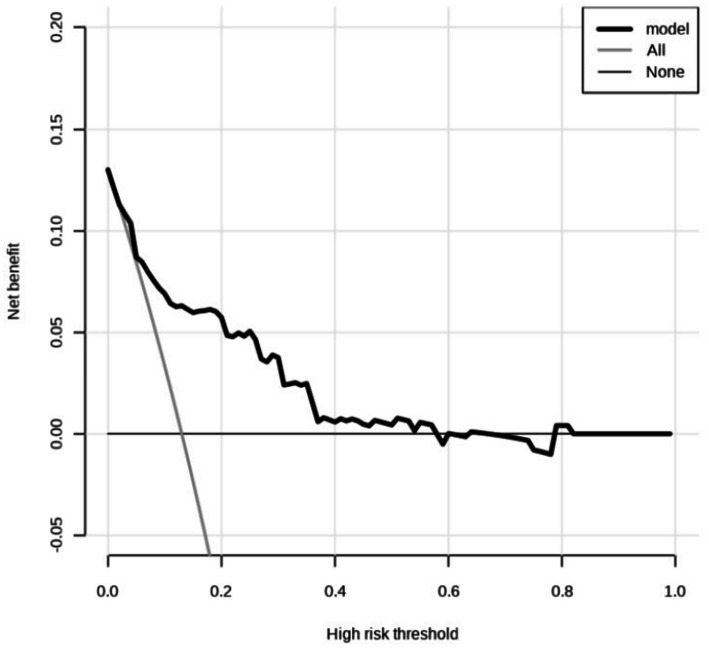
Decision curve analysis. The predicted probability range is 0.02 to 0.81. The horizontal line indicates that the net returns of all factors are zero. The dashed line indicates that all candidate factors have an impact and interventions have been made for all factors. The above curve was compared with net income (a negative sloping back slash).

Decision curve analysis (DCA) was conducted to evaluate the clinical prediction performance of a model combining NIHSS score immediately after thrombolysis, stroke TOAST classification, and diabetes for diagnosing END after thrombolysis. The results demonstrate that the model yields superior clinical benefit when the threshold probability ranges from 0.02 to 0.81, suggesting its potential clinical predictive value (Figure 4).

## Discussion

In this investigation, we initially identified the risk factors associated with early neurological deterioration (END) following intravenous thrombolysis for acute ischemic stroke (AIS) through univariate analysis, subsequently employing multivariate logistic regression analysis. The findings indicated that an elevated NIHSS score immediately following thrombolysis was significantly and positively associated with END occurrence and served as a stronger predictor than the pre-thrombolysis NIHSS score. Additionally, the large artery atherosclerosis subtype (TOAST classification) and a history of diabetes were also identified as independent predictors of END. Utilizing these identified risk factors, we constructed a nomogram that achieved an area under the curve (AUC) value of 0.809, demonstrating a significant clinical predictive capability for the occurrence of END. Different from previous studies, the model incorporated readily accessible predictive variables obtained from routine clinical examinations (9, 10). Our study demonstrated that the NIHSS score immediately after thrombolysis was a stronger predictor of early neurological deterioration (END) than the pre-thrombolysis NIHSS score. The post-thrombolysis NIHSS assessment more accurately reflects both the efficacy of rt-PA thrombolysis and the extent of reperfusion in the ischemic penumbra. Consequently, it holds greater clinical utility for prognosticating patient outcomes and anticipating END.

Clinical experience indicates that early neurological deterioration (END) following intravenous thrombolysis for acute ischemic stroke can notably raise both mortality and disability rates among patients, placing a substantial burden on families and society. However, the underlying causes of END remain poorly understood, and there is currently no method for early detection. Consequently, identifying the risk factors associated with END, providing timely interventions for high-risk patients, and developing effective diagnosis and treatment strategies are anticipated to lower the occurrence of END and enhance patient outcomes.

The definition of END remains inconsistent because of variations in the time frame and evaluation criteria. In clinical practice, it is commonly accepted that an increase of ≥4 points in the NIHSS score from the baseline within 24 h following thrombolytic therapy, or death, serves as the diagnostic criterion for END (11). A meta-analysis encompassing 29 studies on early neurological deterioration (END) following recombinant tissue plasminogen activator (rtPA) thrombolysis, which included a total of 65,960 patients, indicated that the incidence of END within a 24-h period was approximately 14% (95% confidence intervals (CI), 12–15%) (12). In our investigation, the observed incidence of END was approximately 12.9%, which aligns closely with the findings of prior research.

The NIHSS score is widely utilized in clinical settings to assess the extent of neurological impairment in patients with AIS. An elevated NIHSS score immediately following thrombolysis is indicative of a severe stroke and a suboptimal thrombolytic response, which may be associated with complications such as hemorrhagic transformation and malignant cerebral edema resulting from extensive infarction, thereby contributing to the onset of END (13–15). In this investigation, the median NIHSS score following thrombolysis in the non-END group was recorded at 4 points, in contrast to a score of 16 points in the END group. This disparity suggests that patients exhibiting more pronounced neurological deficits are at an increased risk of developing END. Furthermore, a separate study examining functional outcomes 3 months post-intravenous thrombolysis identified the NIHSS Score immediately after thrombolysis as an independent risk factor associated with unfavorable prognoses (15). The NIHSS score serves as a valuable tool for assessing and quantifying the severity of AIS, and it has been associated with patient prognosis. However, its time-dependent nature necessitates repeated evaluations of neurological function, particularly before and after thrombolysis, throughout the treatment course. Monitoring for changes in neurological status and incorporating multiple risk factors can help identify AIS patients at risk of END.

The TOAST classification represents a widely utilized framework for categorizing strokes in clinical settings. It classifies ischemic strokes into five distinct categories: large artery atherosclerosis, cardioembolic, small artery occlusion, and other or unexplained types. Numerous studies have indicated that large artery atherosclerosis is the predominant type of stroke and is significantly associated with the incidence of early neurological deterioration (END) following thrombolytic therapy (16, 17). Our study revealed a significantly higher prevalence of large artery atherosclerosis in the END group (53.1%) compared to the non-END group (22.6%) (*p* < 0.05). Multivariate analysis identified TOAST subtype of large arterial atherosclerosis (LAA) as an independent risk factor for END (OR = 2.65, 95% CI: 1.13–6.22, *p* = 0.025). Patients with large artery disease often present with a greater thrombus burden, potentially leading to hemodynamic instability, increased perfusion damage, and difficulty establishing collateral circulation, thereby increasing the likelihood of END. Intravenous thrombolysis may achieve a lower recanalization rate in cases of large thrombus burdens, particularly in large artery lesions or tandem lesions, potentially resulting in emboli detachment and distal vessel occlusion, raising the risk of symptomatic intracranial hemorrhage or bleeding at other sites. Similar findings were reported in a retrospective study of 210 patients by KIM et al., which found that 57 patients (26.2%) experienced early neurological deterioration. This study suggested that TOAST subtype of large arterial atherosclerosis (LAA) is a predictor of ischemia progression within 24 h after thrombolysis and an independent risk factor for early neurological deterioration (18).

Our investigation revealed that 14 patients with END had a history of diabetes, representing 43.8% of the cohort, while 50 patients without END had a history of diabetes, accounting for 23%. The prevalence of diabetes in the END group was significantly higher than that in the non-END group (*p* = 0.012). Multivariate analysis indicated that diabetes serves as an independent risk factor for END following thrombolysis, corroborating findings from prior studies (18, 19). The underlying mechanism contributing to END may involve the ischemic and hypoxic conditions present in the infarct center and the surrounding ischemic penumbra following an acute ischemic stroke, which results in a lack of glycolysis. Elevated blood glucose levels can lead to increased lactate production in cerebral tissue, adversely affecting the metabolism of cells in areas of low perfusion, resulting in cellular edema and an expansion of the infarct zone. Prolonged hyperglycemia may compromise the integrity of the blood–brain barrier, thereby heightening the risk of spontaneous intracranial hemorrhage. Additionally, the vascular damage observed in diabetic patients diminishes their regulatory capacity and tolerance, complicating the establishment of new collateral circulation. Consequently, it is imperative to implement early blood glucose monitoring, manage diabetes effectively, and maintain appropriate blood glucose levels in the treatment of stroke patients (10, 20, 21). Therefore, early blood glucose monitoring, treatment of diabetes, and reasonable blood glucose control are essential in the treatment of stroke patients.

This study presents several limitations. Firstly, all participants were sourced from the People’s Hospital Affiliated with Jiangsu University, which may affect the generalizability of the findings. The limited sample size also may constrain the statistical robustness of the results. Secondly, the baseline imbalances between the END and non-END groups, though adjusted via multivariate regression, may still influence the model’s generalizability. Future multi-center studies with larger cohorts are warranted to validate these findings using propensity score matching or stratified analyses. Thirdly, this study involved multiple statistical comparisons, which may increase the risk of type I error. No correction for multiple comparisons was applied, and this should be considered a limitation. Fourthly, although the Hosmer–Lemeshow test was used to assess model calibration, it does not reflect predictive accuracy. Internal validation was limited, and no bootstrapping or external validation was performed, which may affect the generalizability of the model. Fifthly, as patients that received EVT were excluded from the analysis, the findings of the study cannot be generalized to them. Lastly, as this investigation serves as a preliminary exploratory study, we did not perform additional experiments to categorize the imaging characteristics of stroke patients. Future studies will involve more comprehensive evaluations that integrate additional clinical data, such as MRI and CT findings, to establish a multimodal predictive model. This approach will enhance our understanding of the risk factors associated with END in patients with AIS undergoing intravenous thrombolysis.

This study aimed to develop a prognostic model to identify patients at risk of early neurological deterioration (END) after intravenous thrombolysis, rather than to determine causality or guide changes in clinical practice. While some predictors may be unmodifiable, early risk identification can help optimize monitoring and supportive care. We also clarified that patients with hemorrhagic transformation or who received endovascular treatment were excluded, and the cohort represents only those treated with intravenous thrombolysis. This helps ensure that the findings reflect prognostic not causal associations.

## Conclusion

In conclusion, our study identifies NIHSS score immediately after thrombolysis, history of diabetes, and large artery atherosclerotic stroke (TOAST classification) as independent risk factors for early neurological deterioration (END) following intravenous thrombolysis in acute ischemic stroke patients. Early recognition of these factors may guide clinical vigilance and inform targeted interventions to mitigate END risk.

## Data Availability

The original contributions presented in the study are included in the article/supplementary material, further inquiries can be directed to the corresponding author.
